# Effect of Marginal Bone Integrity and Aftermarket Abutment Screws on Dental Implant Systems—A Preliminary Study with Finite Element Method

**DOI:** 10.3390/ma15175952

**Published:** 2022-08-28

**Authors:** Yu-Ling Wu, Ming-Hsu Tsai, Hung-Shyong Chen, Ching-Ping Lin, Aaron Yu-Jen Wu

**Affiliations:** 1 Department of Dentistry, Kaohsiung Chang Gung Memorial Hospital and Chang Gung University College of Medicine, Kaohsiung 833, Taiwan; 2 Department of Mechanical Engineering, Cheng Shiu University, Kaohsiung 833, Taiwan; 3 Center for Environmental Toxin and Emerging-Contaminant Research, Cheng Shiu University, Kaohsiung 833, Taiwan

**Keywords:** aftermarket screw, non-original screw, marginal bone loss, stress distribution, finite element analysis

## Abstract

Bone resorption around implants is quite common, and the maturity and popularization of computer-aided design and computer-aided manufacturing (CAD/CAM) technology have made the use of aftermarket abutment screws more widespread. This study aimed to explore the biomechanical influence of these two common factors on the internal stress of an implant system using three-dimensional finite element analysis (3D FEA). The FEA results indicated that under the same loading conditions, the use of an aftermarket screw had the greatest impact on the screw itself among the three components of the implant system, while the maximum stress increased by 6.3% and 10.5% in the bone integrity and bone loss models, respectively. Moreover, the marginal bone loss models had the greatest impact on the implant fixture, with a maximum stress increase of 51.8% on average. Evidently, the influence of bone loss might be far greater than that of the aftermarket screw; however, any factor could be enough to cause clinical failure. Therefore, we should pay more attention to the maintenance of the long-term peri-implant marginal bone integrity.

## 1. Introduction

A dental implant is considered a predictable treatment to replace a missing tooth [1,2]. The implant assembly is composed of an implant fixture and prosthetic components, including a crown, abutment, and fixation screw, and is osteointegrated into alveolar bone to exercise its chewing function. With advancements in computer-aided design and computer-aided manufacturing (CAD/CAM) techniques in dentistry, clinicians can now select prosthetic components from different manufacturers, including the original equipment manufacturer (OEM) [3] or aftermarket manufacturers. Aftermarket manufacturers can replicate the design of prefabricated OEM implant prosthetic components and produce low-cost CAD/CAM-engineered components for clinical use with high efficiency [4]. However, there are currently very few studies that discuss the biomechanical effect of aftermarket prosthetic components on the whole dental implant system.

Avoiding complications and achieving long-term success in dental implantation is important in our aging society, so the maintenance of the bone around dental implants is of concern because the amount of peri-implant marginal bone loss is directly related to delayed implant failures [5,6]. In many situations, marginal bone loss occurs even when biomechanical variables are controlled, because the etiology of bone loss is multifactorial [5].

Based on a previous study by Albrektsson et al. [7], it has been widely accepted that a peri-implant marginal bone loss in the order of 1 mm during the first year of prosthetic loading, and an annual bone loss thereafter not exceeding 0.2 mm, is a natural feature and consistent with successful treatment. However, since then, new implant surfaces and new implant brands, different implant-platform connections, and different time frames between surgical placement procedures and prosthetic occlusal loading have continuously been introduced. Thus, maintaining peri-implant marginal bone integrity can be achievable in a daily clinic today. Currently, an important issue is the management of preventing peri-implant bone resorption caused by, for instance, occlusal overload or/and peri-implantitis. This means that the key factor for long-term implant success is maintaining the initially achieved peri-implant marginal bone level as coronally as possible [6].

Excessive marginal bone loss can affect the biomechanical anchorage of implants, increasing the length of the lever arm and, consequently, increasing stress in the adjacent structures [8,9]. However, the biomechanical effect of marginal bone loss on the components of the dental implant assembly has not been widely discussed, especially with respect to the screw, which is a fragile and critical connection that affects the success or failure of the whole implant assembly.

A complication in the screw could be a serious issue. The screw is the smallest component in the entire implant assembly, and it connects the abutment to the implant fixture. Screw loosening and fracture complications can be as high as 45% over a 10-year period. Such loosening is an indicator of an inadequate biomechanical design and/or occlusal overload [10,11].

One important mechanical factor that prevents abutment screw loosening and fracture is screw joint preload. When the screw is tightened, there is a tension force generated between the abutment and the inner connector of the implant along the screw thread surfaces; this force is a direct determinant of clamping force [12]. The preload is related to the microstructure and mechanical properties of the titanium alloys of the screw. The clinical performance of CAD/CAM titanium screws can be negatively affected, if aftermarket manufacturers use raw materials that differ from those used by the original manufacturer and if they fail to follow the machining guidelines [13]. However, few studies have examined aftermarket abutment screws, and their clinical effect has not yet been completely determined.

Therefore, the purpose of the present study was to evaluate the biomechanical effect of implants and prosthetic components with different screw manufacturers in the presence of bone loss by using three-dimensional (3D) finite element analysis (FEA).

## 2. Materials and Methods

**Figure 1:**
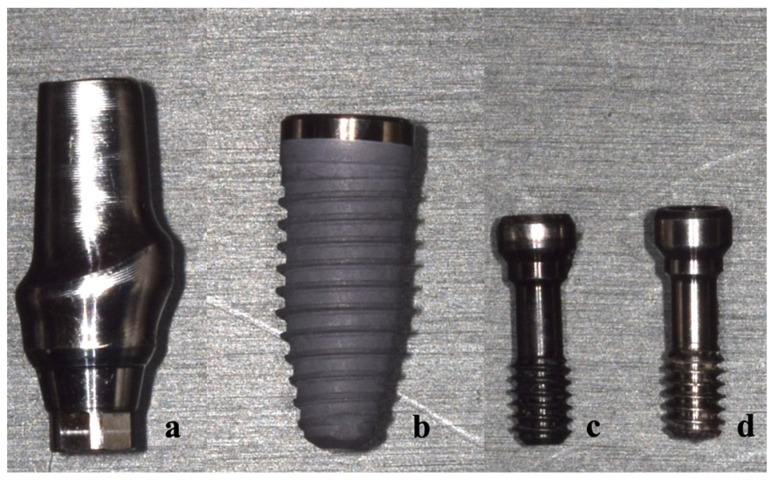
Components of the abutment–screw–implant system: (**a**) titanium abutment, (**b**) implant fixture, (**c**) OEM abutment screw, and (**d**) aftermarket abutment screw.

A NobelReplace Conical Connection PMC dental implant (Ø = 4.3 mm × 10 mm, Nobel Biocare, Gothenburg, Sweden) and an esthetic Conical Connection RP abutment (Nobel Biocare, Gothenburg, Sweden) with a zirconia crown were used to create a morphology resembling the right central incisor. Fixation screws, including OEM abutment screws (Nobel Biocare, Gothenburg, Sweden) and aftermarket CAD/CAM abutment screws (JingGang, Tainan, Taiwan), were obtained from two manufacturers. The aftermarket manufacturer fabricated the screws based on data for OEM abutment screws obtained from an optical scanning machine. The aftermarket manufacturer input the information into their own CAD software and made adjustments based on experience before finally milling the screw with the CAM system. According to previous investigation [4], significantly different measurements of screw lengths (including head length, neck length, shank length, and thread length) and screw angles (hex angle and thread angle) were obtained for the OEM and aftermarket CAD/CAM screws by using a 3D microscope measurement (Figure 1).

**Figure 2:**
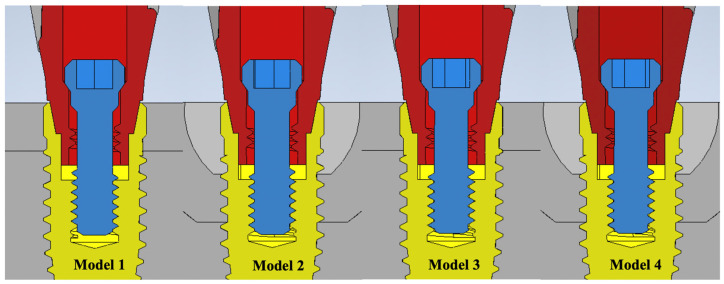
The cross-section of four CAD geometry models. Model 1—OEM screw with no bone loss; Model 2—OEM screw with 3 mm angular bone loss; Model 3—aftermarket CAD/CAM screw with no bone loss; Model 4—aftermarket CAD/CAM screw with 3 mm angular bone loss.

**Figure 3:**
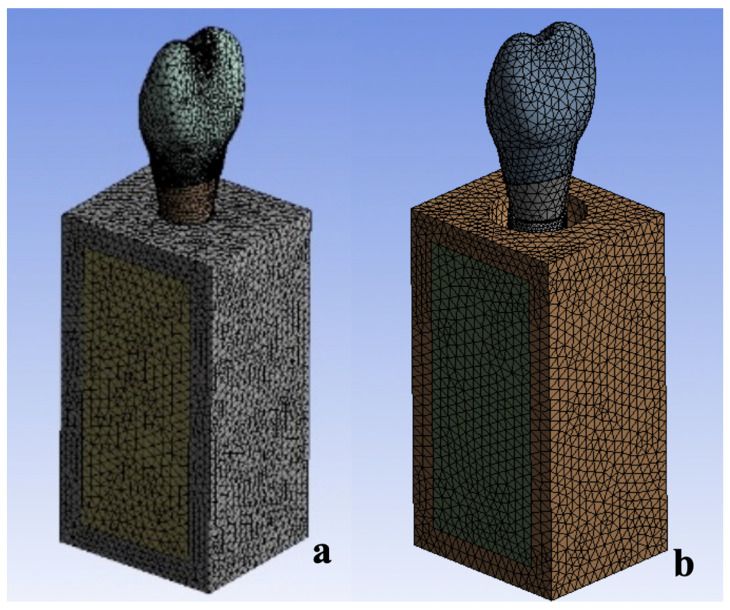
The finite element models (the mesh is shown) of the implant and surrounding bone. (**a**) Models 1 and 3. (**b**) Models 2 and 4.

Four models with different screws and variable marginal bone levels were used in this analysis (Table 1); the CAD models were developed with CAD system (Inventor 2017, Autodesk Inc., San Rafael, CA, USA) using 3D microscope measurements (Figure 2). Models 1 and 2 consisted of an implant fixture, titanium abutment, and OEM screw. Models 3 and 4 consisted of an implant fixture, titanium abutment, and aftermarket CAD/CAM screw. The abutment–screw–implant systems were placed into a bone block model of size 25 mm × 12 mm × 10 mm. The bone block model consisted of 2 mm cortical bone with a spongy center [14]. Models 1 and 3 had normal bone tissue levels, while Models 2 and 4 exhibited peri-implant angular bone resorption with a depth of 3 mm [15,16]. All components were meshed with triangular elements (Figure 3), and each model comprised a similar number of elements (Table 1).

After the modeling phase, the models were exported to an FE analysis program (ANSYS Workbench, version 14.5, Swanson Analysis Systems, Elisabeth, PA, USA) for processing. The implant, abutment, and screws were assumed to be homogeneous with isotropic elastic properties [17]. The material properties of the implant components and bone were established based on the results of previously published studies (Table 2) [18,19,20]. Using energy-dispersive X-ray spectroscopy (JSM-6360, JEOL, Akishima, Japan), the materials of the dental implant were confirmed to be made of pure titanium, while the abutment, OEM screw, and aftermarket CAD/CAM screw were confirmed to be made of TiAl6V4.

All interfaces between the screw–abutment–implant system and bone were set as contacts. The frictional coefficients of the abutment–screw, screw–implant, and abutment–implant interfaces were assumed to be 0.3 [21], and the frictional coefficients of the surface contacts of the rough implant surface with the cancellous bone and cortical bone were assumed to be 0.77 [22] and 0.65 [23], respectively.

**Figure 4:**
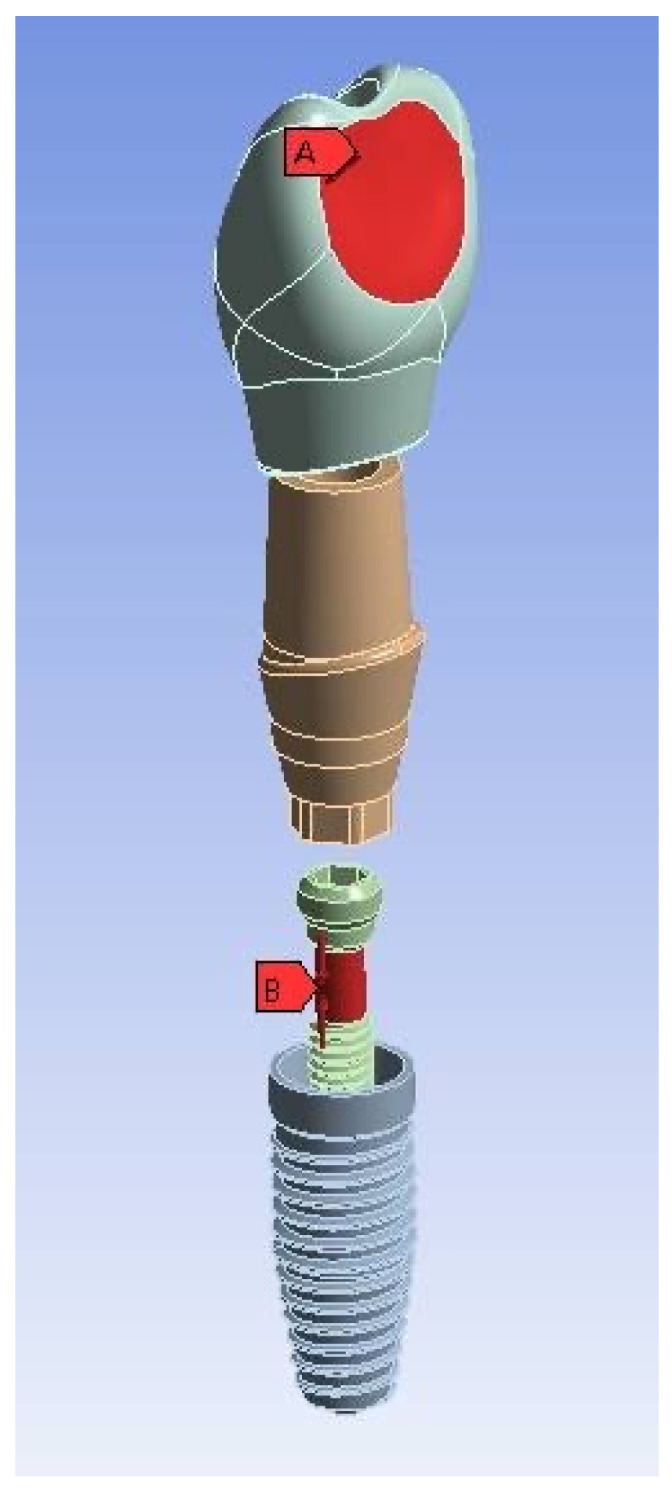
The exploded view of the crown-abutment-screw–implant assembly and loading direction: (**A**) lateral force of 170 N applied to the palatal surface of the crown and (**B**) axial force of 511 N applied to the screw.

A lateral force of 170 N was applied on the whole palatal surface of the crown at 45° relative to the long axis of the implant fixture [24]. For screw tightening, a 35 N·cm torque was applied, and an axial force—which was calculated as 511 N—was created [4] (Figure 4).

Four models with the OEM and aftermarket screws and variable bone levels (no bone loss or 3 mm angular bony defect) were used to investigate the stress distribution in the screw–abutment–implant connection system. For a direct and systematic comparison, the same boundary conditions, constraints, and load conditions were applied to the models. ANSYS Workbench (version 14.5, Swanson Analysis Systems) was installed on a computer to analyze the model data and perform a stress analysis of the implant assembly when subject to an oblique periodical loading [14].

## 3. Results

Under the same loading conditions, the maximum von Mises stress analysis showed that peri-implant bone loss increased tensile stress in the implants and prosthetic components. The stress distributions of Models 1 to 4 were similar, where the maximum von Mises stress occurred on the implants, followed by the screws and titanium abutments. The maximum stress values and strength conditions were listed in Table 3.

**Figure 5:**
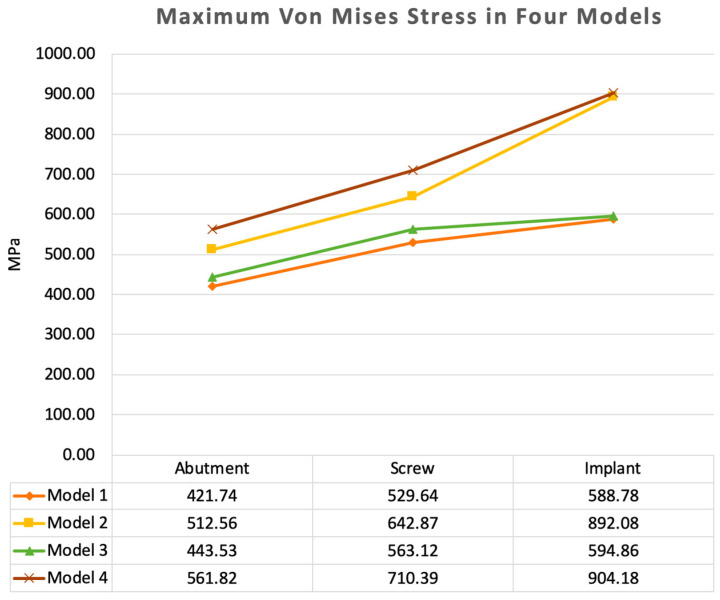
Maximum von Mises stress values for all the components of the models. Marginal bone loss and aftermarket screws were both were associated with an increase in the stress in the implants and prosthetic components.

In the OEM screw groups (Model 1 compared with Model 2), the maximum stress on the implant was 588.78 MPa in Model 1 and 892.08 MPa in Model 2, or an increase of 51.5% in the bone loss model. The stress on the screw increased from 529.64 MPa to 642.87 MPa, respectively, or an increase of 21.4% in the bone loss model. The stress on the abutment increased from 421.74 MPa to 512.56 MPa, respectively, or an increase of 21.5% in the bone loss model. Bone loss caused the implant stress in the aftermarket screw group (Model 3 compared with Model 4) to increase from 594.86 MPa to 904.18 MPa, an increase of 52.0%. Furthermore, the screw stress increased by 26.2% from 563.12 MPa to 710.39 MPa, while the abutment stress increased by 26.7% from 443.53 MPa to 561.82 MPa (Figure 5).

In the intact marginal bone models, the maximum stress values at the abutment, screw, and implant in Model 3 (aftermarket screw) were 5.2%, 6.3%, and 1.0% larger than those in Model 1 (OEM screw), respectively. In the marginal bone loss models, the maximum stress values at the abutment, screw, and implant in Model 4 were 9.6%, 10.5%, and 1.4% larger than those in Model 2, respectively.

**Figure 6:**
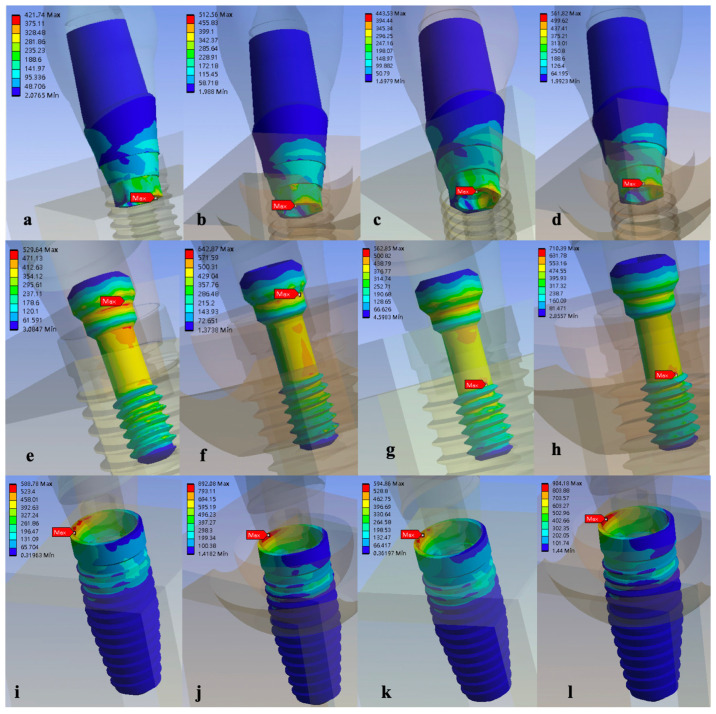
Stress distribution and maximum von Mises stress of the four models under the same loading conditions of (**a**) Model 1, (**b**) Model 2, (**c**) Model 3, and (**d**) Model 4; in the screw of (**e**) Model 1, (**f**) Model 2, (**g**) Model 3, and (**h**) Model 4; and in the implant of (**i**) Model 1, (**j**) Model 2, (**k**) Model 3, and (**l**) Model 4.

An analysis of the location of maximum stress in the implants of all groups showed that the maximum stress was concentrated at the buccal side of the implant wall. In the abutment of all groups, the maximum stress was concentrated at the palatal side of the abutment neck (the connection where the abutment was inserted deep into the implant). In the screws, the position of maximum stress changed from the palatal side of the first step of the screw head for the OEM screw (Model 1 and 2) to the junction of the screw shank and thread for the aftermarket screw (Model 3 and 4) (Figure 6). 

## 4. Discussion

Aftermarket prosthetic components have been widely used in recent years due to advancements in CAD/CAM techniques in dentistry. However, most studies have focused on aftermarket abutments with OEM screws. Chang et al. [25] compared the fracture resistance of different types of CAD/CAM zirconium abutments, including OEM and aftermarket, and their results showed no difference between the two. However, in the majority of the literature, whether during in vitro mechanical experiments or clinical studies, it has been reported that aftermarket abutments are more likely than OEM abutments to produce poor results in the implant system [26,27,28,29,30,31,32]. Gigandet et al. [26] found that non-original abutments differed in the design of the connection surfaces and material and demonstrated a higher rotational misfit, which may result in unexpected failure modes. Rizvi et al. [27] revealed that original abutments presented more predictable outcomes than non-original abutments, with regards to the parameters investigated, including the precision of fit, microleakage, micro-morphological differences, micro-motion, rational misfit, fracture resistance, etc. In addition, they showed that the external hex system might be more tolerant than the internal hex system, in terms of using aftermarket implant abutments. A similar situation was also reported for veneered zirconium abutments, a new form of abutment, cemented on non-original titanium bases, where not only mechanical but even clinical parameters were affected. Asgeirsson et al. [28], Stucki et al. [29], and Strauss et al. [30] found that non-original titanium bases might influence initial marginal bone loss, without affecting their favorable long-term clinical performance. In terms of the effect of aftermarket abutments with original screws, Alonso-Pérez et al. [31] showed that original abutments exhibited lower percentages of reverse torque reduction after cyclic loading than aftermarket abutments. Hsu et al. [32] found that aftermarket CAD/CAM titanium abutments yielded comparable success and survival rates after 6 years of follow-up, but with relatively high incidence of screw loosening, especially in patients receiving single-implant crowns. In an FEA study, Wu et al. [4] revealed that aftermarket screws suffered higher stress than original screws, and the use of aftermarket screws was associated with unpredictable outcomes. 

In this preliminary study, considering the data presented earlier, minor differences in morphology were found between the original (OEM) and non-original (aftermarket) screws [4]. Even a minor discrepancy could create a great deal of change inside the whole implant assembly. In the experiment with intact marginal bone, the aftermarket screw changed the position of maximum stress in the screw from the screw head to the screw shank (the narrowest region below the implant platform) and increased the maximum stress of its own screw by 6.3% and the stress of the corresponding abutment by 5.2%; however, it had a limited impact on the stress of the implant fixture itself, which increased by 1.0%.

Peri-implant marginal bone loss is currently an important and widely discussed issue in implantology. A variety of treatments have been suggested to preserve implants with excessive bone loss, as is observed with peri-implantitis [33]. Peri-implantitis is an unwanted biological complication that is defined as “a pathological condition occurring in tissues around dental implants, characterized by inflammation in the peri-implant connective tissue and progressive loss of supporting bone” [34]. If the bone loss extends at least 3 mm apical to the implant platform, it will be considered as diseased [34,35]. The prevalence of peri-implantitis ranges from 11.3% to 47.1%, with a cumulative complication rate of 48.03% after a follow-up period of 10–16 years [36,37,38,39]. Bone loss results in a reduction in the contact surface of the implant and surrounding bone; in addition, mechanical loading affects the stress distribution at the interface of the implant and bone and in the implant system. With the loss of peri-implant-supporting bone, patients may have to face the eventual consequence of implant loss. This translates to the loss of quality of life, esthetics, chewing function, and time and causes psychosocial stress on the patients [39,40]. Therefore, the prevention and management of peri-implant bone loss have become the focus of many researchers.

According to the European Federation of Periodontology Workshop [41], the following aspects are recommended for the clinical routine to maintain the long-term peri-implant marginal bone integrity: risk factors of peri-implantitis such as smoking, excess cement remaining, and ill-fitted implant components should be avoided. Both patient- and professional-administered mechanical methods for plaque removal are effective for the reduction in inflammation in the peri-implant tissue. During recall, the dentist must regularly examine the peri-implant tissue, including probing assessments with special emphasis on bleeding on probing, and the patient’s personal oral hygiene should be regular monitored and reinforcement.

In the models with marginal bone loss (Models 2 and 4), the experimental data showed that marginal bone loss had a significant impact on the entire titanium implant assembly, regardless of whether the abutment screw was from the OEM or aftermarket group. The maximum stress in the three components of the implant assembly, including the implant fixture, abutment, and screw, significantly increased by 51.8%, 24.1%, and 23.8%, respectively, on average. Among the components, the wall stress of the fixture was greatly increased, even exceeding its breaking strength (680 MPa), which leads to a risk of fracture. This result is consistent with most of the literature; the only difference is that we found the walls of the implants in Nobel Biocare’s PMC series were subjected to extreme stress. Lemos et al. [42] found that under vertical bone destruction, implants had higher stress and microstrain values than normal, regardless of the quality of the bone and the implant–abutment connection. In addition, their team also found that the internal hex system had lower stress and microstrain values than the external hex system, but only if there was no bone loss. When marginal bone loss occurs and increases, the external hex system is subjected to lateral force, and there is higher stress concentrated on the fixture and screw. This finding is also similar to the results of this experiment. Yenigun et al. [16] found that 3 mm marginal bone loss caused apparent pressure on the narrow implant system and suggested marginal bone resorption magnitude as a crucial biomechanic parameter for determining mechanical behavior. Kitamura et al. [15] found that conical bone resorption had less of a stress effect on an implant system than pure vertical bone resorption. In this experiment, an angular bone defect, which is more common in clinical practice, was used, and the results still showed that it had a considerable impact on the entire implant system. Perez-Pevida et al. [43] found that different morphologies of bony defects affected the strain distribution and amount in an implant system. 

In this preliminary study, bone loss might be associated with an increase in stress in the implants and prosthetic components, especially when using an aftermarket screw. These findings are in agreement with those of previous FEA studies regarding aftermarket abutments [8,42,43,44,45]. When an unfit aftermarket screw encounters another factor of marginal bone loss, compared with the use of an OEM screw in the presence of bone loss, the stress accumulation and destructive force caused by the unfit screw are compounded. As mentioned above, the order of stress amplification could be as follows: in the screw itself, stress increased by 10.5%, while it increased by 9.6% in the corresponding abutment and only 1.4% in the implant fixture itself. To summarize, it was shown that the use of aftermarket screws might have an impact on the internal maximum stress, changing its position in both intact marginal bone and marginal bone that was damaged by 3 mm, which could increase the possibility of clinical screw fracture. However, the destructive power of marginal bone loss transmitted to the implant assembly could be more impactful than the effect of the aftermarket screws, so, when the two factors occur at the same time, the situation could be worse.

The 3D microscope measurements showed that the commercially available aftermarket screws had statistically different lengths and angles compared to the OEM screws [4]; in this situation, the misfit with the abutment may not be visible to the naked eye in clinical use, and no immediate destructive failure would occur. Problems with manufacturer preprocessing during the 3D optical scanning procedure and CAD and postprocessing errors in the accuracy of CAM are possible reasons for the differences in the size measurement of the CAD/CAM screws fabricated by different manufacturers. With further advancement of CAD/CAM technology, there may soon be a good process that can achieve the same precise geometry as the OEM screws, which will prevent misfits with the prosthetic components. 

There are limitations to this preliminary finite element study: the different manufacturers may produce variable aftermarket screws that may have different results, and only one type of implant assembly design was used. Furthermore, the present study employed a simplified bone shape, where the material properties of the bone were assumed to be isotropic and homogeneous, while peri-implant bone loss shape and volume may vary between clinical situations. Therefore, additional in vitro investigation and clinical research with statistical data on the performance of aftermarket CAD/CAM screws with peri-implant marginal bone loss are required to verify their reliability in long-term clinical use, so more attention should be paid to this type of treatment option.

## 5. Conclusions

Although this FEA study this study investigated only one implant system, one aftermarket screw manufacturer, and a simplified bone model, we might conclude that increasing the maximum stress could relate to both peri-implant marginal bone loss and the use of an aftermarket component, but the influence of marginal bone loss might be far greater than that of the aftermarket screw. Therefore, we should pay more attention to the maintenance of long-term peri-implant marginal bone integrity, and should carefully choose prosthetic components in clinical use. Additional clinical research with different implant design systems and materials is required, to help clinicians in managing peri-implant bone loss and avoiding undesirable implant complications.

## Figures and Tables

**Table 1:** Model descriptions and element numbers of the four finite element models.

**Models**	**Screw**	**Bone Tissue Level**	**Elements**
Model 1	OEM screw	No bone loss	110,313
Model 2	OEM screw	3 mm bone loss	124,689
Model 3	Aftermarket screw	No bone loss	113,294
Model 4	Aftermarket screw	3 mm bone loss	118,390

**Table 2:** Materials properties used in this study.

**Material**	**Young’s Modulus/GPa**	**Poisson’s Ratio**	**Yield Strength/MPa**	**Reference**
Cortical bone	13.4	0.30	-	Akca et al. [18]
Cancellous bone	1.37	0.30	-	Akca et al. [18]
Titanium implant	115	0.35	680	Teixera et al. [19]
Titanium alloys (screw, abutment)	110	0.33	795	Pierrisnard et al. [20]

**Table 3:** Strength condition.

**Model**	**Part**	**Material**	**Max. Stresses/MPa**	**Yield Strength/MPa**	**Compliance with the Strength Conditions**
1	Abutment	Titanium alloy	421.74	795	YES
	Screw	Titanium alloy	529.64	795	YES
	Implant	Titanium	588.78	680	YES
2	Abutment	Titanium alloy	512.56	795	YES
	Screw	Titanium alloy	642.87	795	YES
	Implant	Titanium	892.08	680	NO
3	Abutment	Titanium alloy	443.53	795	YES
	Screw	Titanium alloy	563.12	795	YES
	Implant	Titanium	594.86	680	YES
4	Abutment	Titanium alloy	561.82	795	YES
	Screw	Titanium alloy	710.39	795	YES
	Implant	Titanium	904.18	680	NO

## Data Availability

Correspondence and requests for materials should be addressed to A.Y.-J.W.
